# Polyphenol-Enriched Composite Bone Regeneration Materials: A Systematic Review of In Vitro Studies

**DOI:** 10.3390/ijms23137473

**Published:** 2022-07-05

**Authors:** Kamila Checinska, Maciej Checinski, Katarzyna Cholewa-Kowalska, Maciej Sikora, Dariusz Chlubek

**Affiliations:** 1 Department of Glass Technology and Amorphous Coatings, Faculty of Materials Science and Ceramics, AGH University of Science and Technology, Mickiewicza 30, 30-059 Cracow, Poland; cholewa@agh.edu.pl; 2 Department of Oral Surgery, Preventive Medicine Center, Komorowskiego 12, 30-106 Cracow, Poland; maciej@checinscy.pl; 3 Department of Maxillofacial Surgery, Hospital of the Ministry of Interior, Wojska Polskiego 51, 25-375 Kielce, Poland; sikora-maciej@wp.pl; 4 Department of Biochemistry and Medical Chemistry, Pomeranian Medical University, Powstańców Wielkopolskich 72, 70-111 Szczecin, Poland

**Keywords:** bone regeneration, biocompatible materials, polymers, polyphenols

## Abstract

One of the possible alternatives for creating materials for the regeneration of bone tissue supporting comprehensive reconstruction is the incorporation of active substances whose controlled release will improve this process. This systematic review aimed to identify and synthesize in vitro studies that assess the suitability of polyphenolics as additives to polymer-ceramic composite bone regeneration materials. Data on experimental studies in terms of the difference in mechanical, wettability, cytocompatibility, antioxidant and anti-inflammatory properties of materials were synthesized. The obtained numerical data were compiled and analyzed in search of percentage changes of these parameters. The results of the systematic review were based on data from forty-six studies presented in nineteen articles. The addition of polyphenolic compounds to composite materials for bone regeneration improved the cytocompatibility and increased the activity of early markers of osteoblast differentiation, indicating a high osteoinductive potential of the materials. Polyphenolic compounds incorporated into the materials presumably give them high antioxidant properties and reduce the production of reactive oxygen species in macrophage cells, implying anti-inflammatory activity. The evidence was limited by the number of missing data and the heterogeneity of the data.

## 1. Introduction

### 1.1. Rationale

Patients suffering from bone defects struggle not only with aesthetic but also functional problems that often prevent their daily functioning [1,2,3,4,5]. Depending on the type of defect, the range of possibilities includes prosthetic methods, bone block transplants and stimulation of bone regeneration [2,3,6,7,8]. The dynamic development of prosthetics allows the creation of high-strength restorations with satisfactory function and aesthetics [6]. Further research is conducted in order to develop more and more perfect materials for prosthesis [6,9,10]. Nevertheless, both removable solutions and permanent implants are foreign bodies, the presence of which can irritate the patient’s tissues [3,11]. Moreover, many types of prosthetic restorations require adjustments, corrections and even replacements. The seemingly ideal solution in the form of autologous bone block transplants has a serious disadvantage of injuring the recipient site [12]. In addition, larger defects require major surgery, including microvascular anastomoses [8,12].

The above problems inspired the production of bone regeneration materials. Among the various approaches of medicine to the reconstruction of bone tissue deficiencies is the use of special materials supporting cell adhesion, release and attachment of the necessary components of the cell matrix, which enables comprehensive regeneration. Currently, there are numerous requirements for materials for bone tissue regeneration [2,13]. These materials should not only show osteostimulating effects but also support bone regeneration in a multidimensional way, taking into account, inter alia, antibacterial, anti-inflammatory and anti-carcinogenic effects [14,15]. For this purpose, materials are enriched with drugs, especially antibiotics that exhibit this type of activity [15,16]. However, the growing antibiotic resistance and the proven negative effect of antibiotics on the surrounding tissues at the site of implantation implicate the need to search for new active substances with a similar spectrum of activity [16]. Promising candidates are polyphenolic additives [17,18]. Polyphenols (PPhs) are organic chemical compounds consisting of an aromatic ring and at least two hydroxyl groups. They are found naturally in plants, primarily in the peel and seeds of the fruit. Due to their natural origin, easy availability, low cytotoxicity, as well as demonstrated antibacterial, antioxidant, anti-inflammatory and anti-cancer properties, PPh can be used as active substances [17,18].

There is a wide variety of substances for bone regeneration [7,12,18,19,20]. They fall into two categories: grafts and alloplastic materials [7,19]. Transplants are divided into autogenous (self-derived), allogeneic (from another human patient, including a cadaver) and xenogeneic (derived from an animal). Bone substitutes classically consist of one or more ceramic materials [19]. A breakthrough in the field of developing substances for bone regeneration other than transplants is the induction of their functionalization [18,20]. It was possible due to the development of composites for bone tissue regeneration, from which the polymer matrix constitutes a scaffold for the ceramic filler and gives the opportunity to relatively easily modify such material functions as degradation, release or tissue activity [18,20]. In line with these assumptions, numerous proposals for new materials based on both natural and synthetic polymers containing previously used and innovative ceramic fillers have been developed. Review articles of various aspects related to composites designed for bone tissue regeneration purposes were published, but no syntheses of PPh enriched materials are available [18].

### 1.2. Objectives

This systematic review aims to identify and synthesize in vitro studies that assess the suitability of PPhs as additives to polymer-ceramic composite bone regeneration materials (CBRM).

## 2. Methods

### 2.1. Eligibility Criteria

The PICOS methodology was adopted to assess the eligibility of the reports for this systematic review and meta-analysis [21]. In line with the development of the acronym, the following inclusion and exclusion criteria were adopted: (P) The research problem was the composition of bone regeneration materials containing osteoconductive material, polymer and polyphenol. Due to different indications for use, the specifics of clinical procedure and the course of healing, reports on CBRMs with components of human and/or animal origin were rejected in this study. (I) The desired intervention was to add any PPh to the CBRM. No exclusion criteria are defined within the domain of intervention. (C) As a comparator, it was considered necessary to present in a given report the test results for a material that differs in its composition only in the absence of PPh addition. Materials with other compositions, e.g., containing an additive other than PPh, were not included in the synthesis, but their inclusion in the report was not a criterion for excluding the entire study. (O) As outcomes, any form of evaluation of one or more of the following material properties was required: mechanical, wettability, cytocompatibility, antioxidant and anti-inflammatory. Reports with missing data for calculating PPh additive performance in one or more of the domains listed were excluded. (S) In vitro studies were included in the synthesis. Reports in languages other than English were not eligible. There was no time frame limit for the publication dates of the source documents. These criteria are summarized in Table 1.

### 2.2. Information Sources

This systematic review was based on search engines: (1) Association for Computing Machinery (ACM); (2) Bielefeld Academic Search Engine (BASE); (3) Google Scholar; (4) U.S. National Library of Medicine: PubMed [22,23,24,25]. The searched databases included, respectively: (1) over 3,000,000; (2) over 278,000,000; (3) about 160,000,000; and (4) over 33,000,000 records [22,23,24,25]. All databases were searched on 16 January 2022.

### 2.3. Search Strategy

The search strategy, consisting of keywords and logical conjunctions, was as follows: (composite OR composites) AND (bone OR bones OR osteogenesis OR osteogenic OR osteoinduction OR osteoinductive OR osteoconduction OR osteoconductive OR osteoregeneration) AND (polyphenol OR polyphenols OR tannin OR tannins OR phenylpropanoid OR phenylpropanoids OR flavonoid OR flavonoids)

Due to the differences in the operation of individual search engines, it was necessary to adjust the strategy for each database. Specific search queries for individual databases of scientific articles are presented in Table A1 in Appendix A.

### 2.4. Selection Process

The subsequent stages of study selection followed the methodology of Preferred Reporting Items for Systematic Reviews and Meta-Analyses (PRISMA) [26]. After searching the aforementioned databases, the obtained records were entered into the Rayyan tool (Qatar Computing Research Institute, Doha, Qatar and Rayyan Systems, Cambridge, MA, USA) [27]. With the use of this tool, deduplication processes were performed, initially automatic, then manual, based on suggestions from the application. Subsequently, two researchers (K.C. and M.C.) blindly screened the abstracts. The convergence of the researchers’ assessments was performed using the Cohen’s kappa coefficient. In the event of inconsistency in the ratings, the record was processed to the next stage. Then, the full texts of articles were obtained, and in the case of the lack of such a possibility, this fact was noted. In the next step, the same researchers conducted a blind full-text evaluation. In the event of discrepancy in the assessments, they made the final decision together, and this fact was noted.

### 2.5. Data Collection Process

The extraction of data from individual reports was carried out independently by two researchers (K.C. and M.C.) who then confronted the obtained results and, in case of discrepancies, verified them using the content of the report. The entire data extraction was performed without the use of automation tools. The Google Sheets software (Google LLC, Mountain View, CA, USA) was used to manage the obtained data.

### 2.6. Data Items

The following data were collected from each of the reports: (1) the name of the first author; (2) date of publication; (3) the name of the polymer used; (4) whether the polymer used was of natural or synthetic origin; (5) the name of the filler used; (6) the name of the PPh incorporated into the composite; (7) the PPhs group to which this particular compound belongs; (8) other additional composite components (if applicable); (9) the spatial structure of the composite (e.g., scaffold, film). The above-mentioned outcomes were analyzed only qualitatively, and in the absence of data in any of the domains, the field in the collective table was left blank, and at the synthesis stage, a given report was not taken into account in this specific domain.

For the purpose of the quantitative analysis, the following data were extracted: (10) composite compressive strength; (11) wettability; (12) mass changes during the degradation process; (13) releasing the filler particles in a degradation process; (14) degradative release of PPh; (15) pH change in the course of degradation; (16) viability of cells during incubation with materials enriched with PPh; (17) proliferation of cells; (18) alkaline phosphatase activity of osteoblasts; (19) anti-flammability of the material; (20) antioxidant activity. In the absence of numerical data in a given domain, this was indicated with an empty field in the table for a given synthesis.

### 2.7. Effect Measures

Due to the different methods of measuring the value of quantified domains, the result of relative effectiveness in a specific domain was taken as outcomes, in accordance with the formula

e = t/c × 100%,

where “e” is the effectiveness (expressed as a percentage), “t” is the outcome value for the samples tested expressed in the units adopted by the report authors, and “c” is the analogous value for the control sample, i.e., the composition differs only in the lack of PPh additive. The above formula is the authors’ method developed for the purposes of this work.

### 2.8. Synthesis Methods

Data were synthesized by entering them into collective tables for individual domains. For each of the syntheses, all tests containing numerical data consistent with the accepted outcomes of this review were qualified. Data that were expressed in units that could not be compared even after conversion were rejected. The conversions were performed only within the International System of Units (SI) units, so as to present the data in the most commonly used units, e.g., kPa were converted to MPa. Missing data were indicated with empty boxes in the tables.

## 3. Results

### 3.1. Study Selection

A total of 447 records were identified, most of them from BASE and PubMed. A detailed distribution of entries from individual databases is presented in Table A2. The automatic deduplication tool excluded triplets of 3 and doublets of 43 items. After the automatic rejection of multiple records, the total number of entries dropped to 397. Of the 141 possible duplicates to be checked manually, the authors of this review deleted 85, resolved 53 and designated 3 as non-duplicates. The whole of the two-stage deduplication process ended with the number of 313 unique records, which were subjected to further stages of selection.

**Figure 1:**
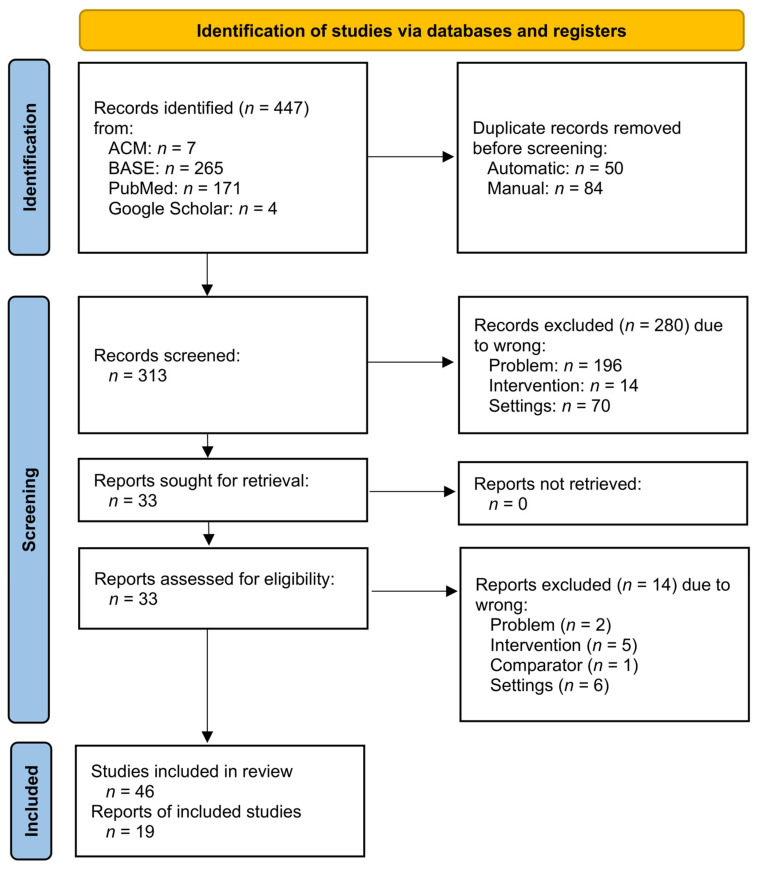
PRISMA flow diagram.

During the abstract screening, 280 items were unanimously excluded, and 33 remained, of which 5 due to a conflict of judges’ decisions. Only the first researcher wanted to include 2, and only the second 3 of the records. Thus, at the screening stage, 98.4% agreement was achieved, which, expressed by Cohen’s kappa coefficient, was κ = 0.91. At the full-text evaluation stage, the judges were in full agreement and rejected 14 subsequent articles. The entire selection process is presented graphically in Figure 1.

### 3.2. Study Characteristics

In total, 19 reports qualified for the review, of which 9 concerned composites based on natural polymers (Table 2), and 10 concerned composites containing synthetic polymers (Table 3). Each of the publications contained comparisons of the reference composite with materials, differing only in the presence of the PPhs additive. Most of the reports described more than one tested material, differing in the composition or method of PPh enrichment. Therefore, in this review, in total, the results of research on 67 materials were synthesized. Due to the numerous results of various characteristics, it was decided to use for the purposes of this article abbreviations describing each of the materials. These abbreviations are listed in Table A3.

### 3.3. Results of Individual Studies and Syntheses

#### 3.3.1. Mechanical Properties

The most frequently tested mechanical property of the composites described in the discussed studies was the compressive strength. The results obtained by individual authors for the subsequent composites are presented in Table S1. In each instance, the first row for a given report presents the values for the reference composite without PPh addition, and the subsequent rows show various PPh additives used in the tests described in this report. In total, all reports proposed 9 non-loaded composites and 25 composites modified with PPhs with measured compressive strength. In 16 cases, the enrichment of materials with phytochemicals resulted in better mechanical properties in the compressive strength domain. The largest increase was 146% of the reference value and was the result of adding ICT to the PLGA/TCP composite. The remaining seven phyto-modifications turned out to reduce the value of the compressive strength by no more than 22%, i.e., no less than 78% of the initial value. The described decrease in strength also occurred as a result of adding ICT to the PLGA/TCP composite. In these two extreme cases, different but not comparable ICT concentrations were used. In the studies where the compressive strength was not determined, the measurements of mechanical stiffness, mechanical stress and strain and compression modulus were used, the results of which are summarized in Table S2.

#### 3.3.2. Wettability

The CBRMs wettability assessment was not a typical element of the analyzed reports. The residual data on this subject, from only one article, suggest that the values of static water contact angles decrease with increasing concentration of the PPhs additive in the studied range (Table S3).

#### 3.3.3. Degradation of Materials

The tested materials were incubated in solutions simulating the tissue fluid, thus making it possible to assess their degradation under conditions similar to those in which they are to be used. With regard to changes in mechanical properties in the course of degradation, changes in the compressive strength were assessed most often. The values of this strength are given in Table S4, and the change from the reference material before the start of incubation is shown in Table S5. This synthesis included studies from 3 reports, which included 1 reference and 3 test materials each, which gave a total of 12 CBRMs. Due to the different measurement conditions, relative values were taken into account for the comparison, with the initial compressive strength of the reference material taken as 100% in each of the reports. The value of this variable in the group of tested materials increased after 12 weeks of incubation only for PLGA/TCP/MICT (131%) PLGA/TCP/HICT (103%) materials, and even for them, it decreased after the next 4 weeks. The remaining tested samples after 12 weeks in the solution had lower compressive strength than before the degradation process, the lowest being PLGA/TCP/ICT-M and PLGA/TCP/ICT-L (34% each). The general fluctuation trend in this domain was downward.

Another variable taken into account in the course of the composites’ degradation was the mass of their samples. Percentage changes in masses are presented, assuming that the initial masses of both the reference and test samples are reference for each series of measurements and amount to 100% (Table S6). A different presentation methodology in this domain results from the trend generally accepted by the authors of these seven reports. For the 16 subjects and 7 reference materials, there was no common time frame that would allow easy comparison of the results. However, in all but one report, the weight was measured approximately one month (28–30 days) from the start of the measurement. At this point, the weight loss of polyphenolic CBRMs based on PLGA and CS polymers was no greater than 6% and each time less than the same measurement for the reference composite. In the same period, hydrogel and alginate materials lost from 60 to 66% of their initial weight, while in the case of the former, the addition of PPhs reduced the loss by about 1–2%, and for alginates, no differences resulting from phyto-additives were noted.

The change in the pH of the incubation solution over time was reported by authors of two reports. The results in this domain for the analyzed materials are presented in Table S7. Each time, the initial pH was 7.2, and in the course of material degradation in the incubation solutions, it decreased. In both reports, it can be seen that a greater admixture of PPh reduced the drop in pH. Despite the use of the same constituent substances of the composites, the pH values after 12 weeks differed significantly; for one series of tests it was from 6.3 to 6.7, and in the second from 3.6 to 4.3, depending on the concentration of the phytomodifier. These differences coexist with the various incubation solutions used.

The evaluation of PPh release from the tested composites was carried out in two incomparable ways. In most of the reports, it was decided to administer each time the percentage of released PPh; in the others, the concentration values were taken into account (Tables S8 and S9). Due to the very diverse compositions and observation periods, it is not possible to compare all data from this domain. The batch syntheses showed that the composites based on silk fibers released more PPh after 4–5 days in PBS (80–90%) than those based on chitosan (14–29%) and PCL (20–28%). This was still less than the PLGA composites in SBF and the alginate composites in PBS, which after 21 days released 10–12% and up to 1% PPh, respectively. The release of the latter up to 40 days from the start of incubation did not exceed 1% and was not investigated further.

#### 3.3.4. In Vitro Cytocompatibility Evaluation

Cell proliferation in contact with the test material is shown as values in Table S10 and converted to percent changes over time in Table S11. There is a general trend of the addition of PPh to CBRM increasing cell proliferation. This increase, expressed as a change in the optical density of the culture, occurred 5–7 days after the start of the measurement, from 143% for scaffolds with naringin adsorbed into gelatin microspheres and encapsulated into nHA/SF scaffolds to 867% for alginate with HA and naringin. For the first material, as the only one, the maximum value was observed (after 7 days), and a further decrease in the optical density value of the culture (single measurement after 10 days) occurred, which concerned both the reference material and the two subjects containing PPhs.

The anti-inflammatory and antioxidant activities of the materials were assessed by authors of only one report and are presented in Tables S12 and S13, respectively. The addition of PPhs resulted in an improvement of the tested values in each of the three domains discussed; however, the lack of the possibility of a meta-analysis due to the lack of data from the other sources leaves these issues without further comment.

Cell differentiation was most commonly assessed by examining the activity of ALP in the following days. Data on this subject are presented in Table S14. The period of 5–7 days from the start of the measurement, common for all studies, resulted in increases in ALP activity from 123% for SF/SBA15 composite loaded with icariin to 439% for SF/SBA15 with BMP2 composite loaded with icariin compared to just as long incubated reference materials in individual reports. These two extremes were observed in one study using bone marrow mesenchymal stem cells and Dulbecco’s modified Eagle’s medium. In the entirety of the synthesized data, there was no report that the activity of ALP decreased due to the addition of PPhs.

## 4. Discussion

### 4.1. General Interpretation of Results

#### 4.1.1. Mechanical Properties

In most studies, the addition of PPh increased the compressive strength of the material. Additionally, it can be seen that these values depended on the concentration of polyphenolic compounds [39,41,45]. This can be explained by the formation of additional bonds at the interface between the polymer matrix and the filler resulting from the presence of numerous hydroxyl groups in PPhs [47]. The phenolic hydroxyl groups present in polyphenols under certain conditions play the role of donor of hydrogen bonds capable of forming intermolecular structures [17]. The hydrogen bonds formed between the hydroxyl groups ensure cross-linking of the material, thus improving its mechanical properties [17,18]. Additionally, the modification of the polymer with phenolic groups derived from PPh allows for better binding with the filler, which means that polyphenolic compounds play the role of coupling agents. Another phenomenon that can improve the mechanical properties of materials is oxidation of the catechol group. As a result, radical coupling reactions take place, resulting in the formation of covalent bonds with the simultaneous formation of polymerizable catechol-catechol adducts. As a result, a chemical cross-linking reaction takes place, which can lead to a hardening of the material [18]. 

#### 4.1.2. Degradation of Materials

Materials with added PPhs degrade more slowly than the reference materials. This probably results from the improvement of the mechanical properties due to the formation of additional bonds. The additional bonds created at the phase boundary strengthen the material, as evidenced by a slower decrease in the weight of PPhs enriched materials. It can be seen from the studies by Xie et al. (2010) and Chen et al. that as the concentration of PPhs in the materials increases, the loss of mass in the degradation process is slower [45,46]. The studies of Lai et al. and Chen et al. showed that at the end of the assumed degradation period, materials with a higher PPhs content demonstrate greater compressive strength [41,45]. However, the same results show that there are limits to the concentration of PPhs above which further addition of PPhs causes a secondary deterioration of the mechanical properties in the studied domain. The initial drop in the pH of the incubation fluid recorded by Xie et al., (2010) and Chen et al. indicates a process of polymer degradation [45,46]. Acidic hydrolysis products initially lower the pH of the environment [48]. Then, the test results indicate fluctuations in the pH index, which in turn is the result of the exchange of calcium ions between the TCP modified composite and the incubation fluid. The release of calcium ions causes alkalization of the environment, which causes a slight increase in pH [49]. In turn, the slight lowering of the pH is due to the degradation of the polymer matrix. These advantages of the degradation process and the formation of a calcium phosphate layer on the surface of materials are characteristic of this type of biomaterials. The addition of polyphenolic compounds causes a lower initial drop in pH for materials with a higher concentration of PPh. This is another evidence that the degradation process is slowed down by the addition of phytochemicals. Incubation of composites also causes a gradual release of polyphenolic compounds. All researchers observed an initial increase in the level of release followed by stabilized release. Such gradual release of the compounds’ polyphenols is the most advantageous from the point of view of material application in engineering tissue because their beneficial properties (such as antioxidant properties, antibacterial, anti-inflammatory or anti-cancer effect) can be fully used by the body in the process of bone regeneration, and too much polyphenols’ release into the body can also be harmful. A higher concentration of polyphenolic compounds in the composite results in an increased level of release. This may be due to the increased number of polyphenolic compounds on the surface of the materials that are first released into the environment.

#### 4.1.3. Cell Studies

The addition of polyphenolic compounds increased the level of cell proliferation. All naturally occurring flavonoids have three hydroxyl groups, one of which is called the catechol group. Oxidized catechol groups called o-catecholquinone are characterized by high reactivity, thanks to which, as a result of reaction with nucleophilic functional groups (amine and thiol groups of peptides), they form stable interphase covalent bonds with cell surfaces [50]. This reaction is called the Michael addition or Schiff-base reaction [18,51]. In addition, tannin acid and some Flavonoid polyphenols have pyrogallol groups that can bind to nucleophiles, such as amides, thiols in proteins or peptides, to form covalent interactions [18,52]. This means that the presence of this type of group not only supports the cross-linking of the material, improving the mechanical properties, but also improves the bioactive properties of the materials.

The analysis of the bone formation and resorption process is based on marking osteoblast enzymes. The activity-related marker of osteoblast’s biological and bone formation is alkaline phosphatase (ALP). All researchers observed an increase in the level of ALP production in contact with the material enriched with polyphenols. Additionally, along with an increase in the concentration of PPh, the level of the analyzed protein increased. Similar observations were made by Bu et al. who noticed that dried plum polyphenols affect osteoblast activity and the formation of mineralized nodules under normal and inflammatory conditions [53]. Additionally, they significantly increased the activity of ALP [53]. Only Chen et al. (2012) for the highest concentration of PPh recorded a result lower than the results for the lower concentrations [45]. This may be due to the use of too high a concentration of PPh compounds, which could have a negative effect on osteoblast differentiation. Nevertheless, the lack of more data does not allow a generalization of this. 

Implantation into the body of the present body causes inflammation and the production of reactive oxygen species (ROS). This is a normal process, and it helps tissue regeneration. However, the long-term production of ROS by high levels of phagocytic cells may cause the opposite effect—implant rejection, dangerous pathological conditions related to protein and DNA damage, which leads to impaired cell function and even resorption of surrounding tissues [54]. Slika et al. noticed the high antioxidant potential of the polyphenols inhibits the formation of ROS [55]. This is due to the presence of hydroxyl groups on the benzene rings. Antioxidants, by catching the free radical, give away a hydrogen atom or an electron, which is the transfer of a proton, resulting in a stable molecule and radical derived from an antioxidant [54]. Cellular studies conducted by Dziadek et al. (2021) using macrophages have shown that the production of reactive oxygen species is lower when cells are cultured in contact with films enriched with polyphenols [37]. Additionally, Dziadek et al. (2021) studied antioxidant activity of the materials using ABTS and DPPH free radical scavenging assays and ferric reducing antioxidant power (FRAP) test. The addition of polyphenolic compounds significantly improved the radical scavenging capacity against the ABTS ^• +^ and DPPH ^•^ radicals, as well as ferric reducing antioxidant potential (FRAP) of the films. However, it should be noted that the values for composite materials with the addition of polyphenolic compounds are lower than for polymer films. This is due to the ability to bind polyphenol compounds inside the structure of materials, so that less PPh is on the surface of the material. Thus, the addition of polyphenolic compounds to bone tissue regeneration biomaterials gives them cytoprotective and anti-inflammatory properties, which can significantly improve the effectiveness of their applications. 

#### 4.1.4. Wettability

An important feature of biomaterials is hydrophilicity. Numerous studies have shown that hydrophilic surfaces bind to cell adhesion and differentiation [56]. Dziadek et al. noticed that the addition of polyphenolic compounds rich in hydroxyl groups significantly improves the wettability of the materials [37]. Part of the PPh was bound in the material structure, and the rest was on the surface. Hence, numerous hydroxyl groups in contact with the external environment affect the hydrophilicity of materials. This may promote full cell adhesion. In addition, Dziadek et al. created materials in the form of films, which also showed greater hydrophilicity and, additionally, facilitated good cell adhesion, which indicates their potential in the field of tissue engineering [57]. It should also be noted that the AS surface showed better wettability than the GS surface. This is probably due to the evaporation of the solvent as the polyphenolic compounds moved to the upper surface (AS) of the material. This resulted in the exposure of more hydroxyl groups at that surface, which in turn resulted in a lower contact angle at that surface.

### 4.2. Limitations of the Evidence

The numerous missing data in the tables presenting individual syntheses resulted from the various measurement methodologies adopted by the authors of the reports, including, in particular, the variable intervals between them. In each instance, the unique compositions of CBRMs, differing in polymer matrices, ceramic fillers and the type of PPhs added to any of the syntheses, did not allow for averaging the results or presenting them in a graphical form. Thus, such inhomogeneous data could not be subject to any statistical analysis.

### 4.3. Limitations of the Review Processes

The adopted assumptions of this systematic review limited the pool of qualified reports to in vitro studies only. During the selection phase, a number of animal studies were additionally identified that also described the use of CBRM with PPhs supplements. They took into account outcomes different from those sought and could not be compared with the results obtained in this work. However, a separate review should be considered in this regard.

### 4.4. Implications of the Results for Practice, Policy and Future Research

The addition of polyphenolic compounds to composite materials for bone regeneration increases the proliferation of cells in contact with them, which proves the cytocompatibility properties of the analyzed composites [29,30,32,36,39,40,44]. Materials enriched with polyphenolic compounds increase the activity of early markers of osteoblast differentiation, indicating a high osteoinductive potential of the materials [28,31,32,34,36,37,40,44,50]. Polyphenolic compounds incorporated into the materials presumably give them high antioxidant properties and reduce the production of ROS in macrophage cells, implying anti-inflammatory activity [17,37,55].

Many of the features of CBRMs with the addition of PPhs were already thoroughly investigated and summarized in this systematic review. However, the use of composites enriched with polyphenolic compounds in clinical applications requires further research. The above-mentioned results indicate the possibility to bind polyphenolic compounds inside the structure of the CBRMs. Future studies should investigate the stability of PPh–filler bonds as a factor influencing the release of these compounds. For the discussed applications, it is important to maintain the balance between the amount of released PPhs and the rate of material degradation. Low stability of polyphenolic compounds encourages the search for such CBRMs compositions that will slow down this process. On the other hand, research should also focus on identifying and eliminating factors that enhance PPhs degradation. An important direction for further research may be the development of a CBRM with high stability during storage before implantation and in the conditions of the human body.

## Figures and Tables

**Table 1:** Eligibility criteria.

Domain	Inclusion Criteria	Exclusion Criteria
Problem description	Composition of CBRMs	CBRMs of human or animal origin
Intervention description	Addition of PPhs to the CBRMs	-
Comparators description	A material with a composition that differs only in the absence of a PPh additive	-
Outcomes description	The difference in mechanical, wettability, cytocompatibility, antioxidant and anti-inflammatory properties of CBRMs	No data available to calculate the efficacy of PPh additive
Settings	In vitro studies	Reports in languages other than English

**Table 2:** List of reports on composites based on natural polymers.

First Author	Year	Used Polymer	Filler	Polyphenol Compound	Type of Polyphenol Compound	Other Additives	Form of Material
Monavari [28]	2021	Alginate di-aldehyde-gelatin	Mesoporous sio2-cao	Icariin	Flavonoid	-	Hydrogel
Yu [29]	2021	Silk fibroin	Nano-hydroxyapatite	Naringin	Flavonoid	Gelatin microspheres	Scaffold
Liang [30]	2021	Sodium alginate	Hydroxyapatite	Naringin	Flavonoid	-	Scaffold
Zhao [31]	2021	Silk fibroin	Hydroxyapatite	Naringin	Flavonoid	-	Scaffold
Xie [32]	2019	Alginate	Hydroxyapatite	Icariin	Flavonoid	-	Scaffold
Kook [33]	2018	Collagen	Hydroxyapatite	Epigallocatechin gallate	Flavonoid	-	Scaffold
Wang [34]	2017	Silk fibroin	Mesoporous silica (SBA-15)	Icariin	Flavonoid	BMP2	Scaffold
Pan [35]	2016	Chitosan	Nano-hydroxyapatite	Icariin	Flavonoid	Fe_3_O_4_ magnetic nanoparticles	Microcapsules
Fan [36]	2012	Chitosan	Nano-hydroxyapatite	Icariin	Flavonoid	-	Scaffold

**Table 3:** List of reports on composites based on synthetic polymers.

First Author	Year	Used Polymer	Filler	Polyphenol Compound	Type of Polyphenol Compound	Other Additives	Form of Material
Dziadek [37]	2021	Polycaprolactone	Bioglass CaO-SiO_2_-P_2_O_5_	Mainly Rosmarinic acid; extract from sage	Phenolic acids, Flavonoids, Phenolic diterpenes	-	Film
Huang [38]	2021	Polycaprolactone	Mesoporous calcium silicate/calcium sulfate	Quercetin	Flavonoid	-	Scaffold
Guo [39]	2020	Poly(1,8-octanediol-co-citrate)	Hydroxyapatite	Tannin acid	Tannin	Nano-silver particles	Scaffold
Cai [40]	2018	Polyetheretherketone	Mesoporous Mg-Ca-Si	Genistein	Flavonoid	-	Not specified
Lai [41]	2018	Poly(lactic-co-glycolic acid)	Β-tricalcium phosphate	Icariin	Flavonoid	-	Scaffold
Dziadek [42]	2017	Polycaprolactone	Bioglass CaO-SiO_2_-P_2_O_5_	Extract from sweet cherry	Not specified	-	Film
Xie [43]	2015	Poly(lactic-co-glycolic acid)	Tricalcium phosphate	Icariin	Flavonoid	-	Scaffold
Wang [44]	2013	Poly(lactic-co-glycolic acid)	Tricalcium phosphate	Icaritin	Flavonoid	-	Scaffold
Chen [45]	2012	Poly(lactic-co-glycolic acid)	Β-tricalcium phosphate	Icariin	Flavonoid	-	Scaffold
Xie [46]	2010	Poly(lactic-co-glycolic acid)	Β-tricalcium phosphate	Icariin	Flavonoid	-	Scaffold

## Data Availability

All the collected and produced data can be found in this article. The review protocol is presented in its entirety in this article and has not been previously published.

## Supplementary Materials

The following supporting information can be downloaded at: https://www.mdpi.com/article/10.3390/ijms23137473/s1.

## Appendix A

**Table A1:** Search queries.

	Search Query
ACM	[[All: composite] OR [All: composites]] AND [[All: bone] OR [All: bones] OR [All: osteogenesis] OR [All: osteogenic] OR [All: osteoinduction] OR [All: osteoinductive] OR [All: osteoconduction] OR [All: osteoconductive] OR [All: osteoregeneration]] AND [[All: polyphenol] OR [All: polyphenols] OR [All: tannin] OR [All: tannins] OR [All: phenylpropanoid] OR [All: phenylpropanoids] OR [All: flavonoid] OR [All: flavonoids]]
BASE	(composite composites) AND (bone bones osteogenesis osteogenic osteoinduction osteoinductive osteoconduction osteoconductive osteoregeneration) AND (polyphenol polyphenols tannin tannins phenylpropanoid phenylpropanoids flavonoid flavonoids)
PubMed	(composite OR composites) AND (bone OR bones OR osteogenesis OR osteogenic OR osteoinduction OR osteoinductive OR osteoconduction OR osteoconductive OR osteoregeneration) AND (polyphenol OR polyphenols OR tannin OR tannins OR phenylpropanoid OR phenylpropanoids OR flavonoid OR flavonoids)
Google Scholar	allintitle: (composite OR composites) (bone OR bones OR osteogenesis OR osteogenic OR osteoinduction OR osteoinductive OR osteoconduction OR osteoconductive OR osteoregeneration) (polyphenol OR polyphenols OR tannin OR tannins OR phenylpropanoid OR phenylpropanoids OR flavonoid OR flavonoids)

**Table A2:** Number of search results.

Database	Number of Records
ACM	7
BASE	265
PubMed	171
Google Scholar	4

**Table A3:** List of tested materials in individual reports.

Author	Year	Material	Abbreviation
Liang [30]	2021	The hydroxyapatite (HA) and sodium alginate (SA) composite	HA/SA
		The hydroxyapatite (HA) and sodium alginate (SA) composite loaded with naringin (NG)	HA/SA/NG
Monavari [28]	2021	The alginate di-aldehyde-gelatin (ADA-Gel) with mesoporous silica-calcia nanoparticles (MSN) composite	ADA-Gel/MSN
		The drug incorporated hydrogel nanocomposite with icariin loaded (ICA) mesoporous silica-calcia nanoparticles	ADA-Gel/ICA-MSN
		The drug incorporated hydrogel nanocomposite with unloaded mesoporous silica-calcia nanoparticles	ADA-Gel/MSN/ICA
Yu [29]	2021	The nanohydroxyapatite (nHA) with silk fibroin (SF)	nHA/SF
		Scaffolds with naringin (NG) encapsulated into nHA/SF scaffold	NG/nHA/SF
		Scaffolds with naringin adsorbed into gelatin microspheres and encapsulated into nHA/SF scaffolds	NG/GMs/nHA/SF
Dziadek [37]	2021	The polycaprolactone (PCL) with bioglass particles (A2)	PCL-A2
		The polycaprolactone with bioglass particles and polyphenolic compounds (1.5 wt.%)	PCL-A2/1.5PPh
		The polycaprolactone with bioglass particles and polyphenolic compounds (3 wt.%)	PCL-A2/3PPh
		The polycaprolactone with bioglass particles and polyphenolic compounds (4.5 wt.%)	PCL-A2/4.5PPh
Huang [38]	2021	The mesoporous calcium silicate calcium sulfate (MSCS) with polycaprolactone (PCL). The ratios of the MSCS/PCL composite 50:50.	MSCS/PCL
		The MSCS/PCL composite loaded with quercetin (Q). The ratios of the Q/MSCS/PCL composite 1:49:50.	MSCS/PCL/Q1
		The ratios of the Q/MSCS/PCL composite 2:48:50.	MSCS/PCL/Q2
Zhao [31]	2021	The hydroxyapatite (HA) with silk fibroin (SF) composite.	SF/HA
		0.03% concentration of naringin (NG) in SF/HA	SF/HA/0.03NG
		0.05% concentration of NG in SF/HA	SF/HA/0.05NG
		0.1% concentration of NG in SF/HA	SF/HA/0.1NG
Guo [39]	2020	Cross-linking parameters: 80 °C, 3 d, 120 °C, V, 1 d The poly(octamethylene citrate) (POC) with hydroxyapatite (HA) composite	POC-HA/1
		Cross-linking parameters: 80 °C, 3 d, 120 °C, V, 1 d POC-HA modified with tannin acid (TA)	POC-THA/1
		Cross-linking parameters: 80 °C, 3 d, 120 °C, V, 1 d POC-HA modified with tannin acid (TA) 50/50	POC-HA/THA/1
		Cross-linking parameters 100 °C, 3 d, 120 °C, V, 1 d of POC-HA composite.	POC-HA/2
		Cross-linking parameters 100 °C, 3 d, 120 °C, V, 1 d of POC-THA composite.	POC-THA/2
		Cross-linking parameters 100 °C, 3 d, 120 °C, V, 1 d of POC-HA/THA 50/50 composites.	POC-HA/THA/2
		Cross-linking parameters 80 °C, 3 d, 120 °C, V, 3 d of POC-HA composite.	POC-HA/3
		Cross-linking parameters 80 °C, 3 d, 120 °C, V, 3 d of POC-THA composite.	POC-THA/3
		Cross-linking parameters 80 °C, 3 d, 120 °C, V, 3 d of POC-HA/THA 50/50 composite.	POC-HA/THA/3
Xie [32]	2019	The hydroxyapatite/alginate composite.	HA/A
		0, 10−7 mol/L Icariin-loaded hydroxyapatite/alginate composite.	HA/A/ICA7
		0, 10−6 mol/L Icariin-loaded hydroxyapatite/alginate composite.	HA/A/ICA6
		0, 10−5 mol/L Icariin-loaded hydroxyapatite/alginate composite.	HA/A/ICA5
Cai [40]	2018	The mesoporous magnesium-calcium-silicate (mMCS)/polyetheretherketone (PK) composite	mMCS/PK
		mMCS/PK composite loaded with genistein (GE)	mMCS/PK/GE
Kook [33]	2018	The epigallocatechin gallate (EGCG)/duck’s feet collagen (DC)/hydroxyapatite composite (HA)	EGCG/DC/HA
		The 1 μM concentration of EDCG solution poured into 2% collagen solution in exact quantity.	1EGCG/DC/HA
		The 5 μM concentration of EDCG solution poured into 2% collagen solution in exact quantity.	5EGCG/DC/HA
		The 10 μM concentration of EDCG solution poured into 2% collagen solution in exact quantity.	10EGCG/DC/HA
Lai [41]	2018	The poly (lactic-co-glycolic acid)(PLGA) and β-calcium phosphate (TCP) composite.	PLGA/TCP-Lai
		The mass ratio of PLGA to TCP to icariin (ICA) was 80:20:0.16	PLGA/TCP/ICA-L
		The mass ratio of PLGA to TCP to icariin (ICA) was 80:20:0.32	PLGA/TCP/ICA-M
		The mass ratio of PLGA to TCP to icariin (ICA) was 80:20:0.64	PLGA/TCP/ICA-H
Wang [34]	2017	The silk fibroin (SF) and mesoporous SBA15 composite.	SF/SBA15
		The SF/SBA15 composite loaded with icariin.	SF/SBA15IC
		The SF/SBA15 with BMP2 composite loaded with icariin.	SF/BMP2/SBA15IC
		The SF/SBA15 with BMP2 composite loaded with icariin.	SF/BMP2/IC/SBA15
Dziadek [42]	2017	The polycaprolactone and melting bioglass A2 (A2melt) composite loading with extract from leaves.	A2melt/PCL/leaves
		The polycaprolactone and sol-gel bioglass A2 (A2gel) composite loading with extract from leaves.	A2gel/PCL/leaves
		The polycaprolactone and melting bioglass A2 composite loading with extract from fruits.	A2melt/PCL/fruits
		The polycaprolactone and sol-gel bioglass A2 composite loading with extract from fruits.	A2gel/PCL/fruits
Pan [35]	2016	The nano-hydroxyapatite with chitosan (CS) composite.	CS/nHA/Pan
		The CS/nHA composite loaded with icariin.	CS/nHS/ICA-Pan
		The CS/nHA and Fe3O4 magnetic nanoparticles composite loaded with icariin.	Magnetic-CS/nHS-ICA
Xie [43]	2015	The poly (lactic-co-glycolic acid)(PLGA) and β-calcium phosphate (TCP) composite.	PLGA/TCP-Xie15
		The PLGA/TCP composite loaded with icaritin (ICT).	PLGA/TCP/ICT
Wang [44]	2013	The poly (lactic-co-glycolic acid)(PLGA) and calcium phosphate (TCP) composite.	PLGA/TCP-Wang
		The PLGA/TCP composite loaded with icaritin.	PLGA/TCP/ICA
Chen [45]	2012	The poly (lactic-co-glycolic acid)(PLGA) and calcium phosphate (TCP) composite.	PLGA/TCP-Chen
		1 cm^3^ PLGA/TCP + 187.5 μg icaritin (ICT)	PLGA/TCP/LICT
		1 cm^3^ PLGA/TCP + 750 μg ICT	PLGA/TCP/MICT
		1 cm^3^ PLGA/TCP + 1875 μg ICT	PLGA/TCP/HICT
Fan [36]	2012	The nano-hydroxyapatite (HA) with chitosan (CS) composite.	CS/nHA-Fan
		The nano-hydroxyapatite (HA) with chitosan (CS) composite loaded with icariin (IC).	CS/nHA/ICA-Fan
Xie [46]	2010	The poly (lactic-co-glycolic acid)(PLGA) and calcium phosphate (TCP) composite.	PLGA/TCP-Xie10
		74 mg icaritin per 100 g PLGA/TCP	PLGA/TCP/ICT-H
		7.4 mg icaritin per 100 g PLGA/TCP	PLGA/TCP/ICT-M
		0.74 mg icaritin per 100 g PLGA/TCP	PLGA/TCP/ICT-L
