# Supplementary material for: Polyphenol-Enriched Composite Bone Regeneration Materials: A Systematic Review of In Vitro Studies
Source: Int J Mol Sci. 2022 Jul 5;23(13):7473. doi: 10.3390/ijms23137473 (PMC9267334; doi:10.3390/ijms23137473)
Supplement: Supplementary file 1 [file ijms-23-07473-s001.zip › S3_PPh_Tables.pdf]

Table S1. Compressive strength. The compressive strength change means an increase or decrease from the reference value of 100% for the composite without PPh addition.

First author	Year	Composite / Polyphenol	Compressive strength value, MPa	Compressive strength change
Yu	2021	nHA/SF	0.27	
		NG/nHA/SF	0.28	103.70%
		NG/GMs/nHA/SF	0.30	111.11%
Guo	2020	POC-HA/1	231.76	
		POC-THA/1	238.82	103.05%
		POC-HA/THA/1	304.71	131.47%
		POC-HA/2	229.41	
		POC-THA/2	283.53	123.59%
		POC-HA/THA/2	323.53	141.03%
		POC-HA/3	245.88	
		POC-THA/3	278.82	113.40%
		POC-HA/THA/3	302.35	122.97%
Xie	2019	HA/A	0.13	
		HA/A/ICA7	0.12	98.41%
		HA/A/ICA6	0.13	99.21%
		HA/A/ICA5	0.12	98.39%
Kook	2018	EGCG/DC/HAp	3.54	
		1EGCG/DC/HAp	3.63	102.54%
		5EGCG/DC/HAp	3.89	109.89%
		10EGCG/DC/HAp	4.05	114.32%
Lai	2018	PLGA/TCP-Lai	2.18	
		PLGA/TCP/ICA-L	2.28	104.60%
		PLGA/TCP/ICA-M	2.10	96.55%
		PLGA/TCP/ICA-H	2.40	110.34%
Xie	2015	PLGA/TCP-Xie15	3.70	
		PLGA/TCP/ICT	4.00	108.11%
Chen	2012	PLGA/TCP-Chen	5.40	
		PLGA/TCP/LICT	5.78	106.94%
		PLGA/TCP/MICT	7.20	133.33%
		PLGA/TCP/HICT	7.88	145.83%
Fan	2012	CS/nHA-Fan	1.50	

		CS/nHA/ICA-Fan	1.46	97.73%
Xie	2010	PLGA/TCP-Xie10	3.79	
		PLGA/TCP/ICT-H	3.58	94.43%
		PLGA/TCP/ICT-M	2.95	77.78%
		PLGA/TCP/ICT-L	3.37	88.89%

Table S2. Results of mechanical parameters other than compressive strength. The change means an increase or decrease from the reference value of 100% for the composite without PPh addition.

First author	Year	Material	Parameter	Value	Unit	Change
Monavari	2021	ADA-Gel/MSN	Mechanical stiffness	54.55	kPa	
		ADA-Gel/ICA-MSN		163.64	kPa	300.00%
		ADA-Gel/MSN/ICA		218.18	kPa	400.00%
Huang	2021	MSCS/PCL	Mechanical stress and strain	6.33	MPa	
		MSCS/PCL/Q1		10.40	MPa	164.30%
		MSCS/PCL/Q2		11.88	MPa	187.68%
Zhao	2021	SF/HA	Compression modulus	0.32	MPa	
		SF/HA/0.03NG		0.32	MPa	100.63%
		SF/HA/0.05NG		0.32	MPa	101.27%
		SF/HA/0.1NG		0.34	MPa	106.33%

Table S3. Static water contact angle of air surface (AS) and glass surface (GS) of materials.

First author	Year	Material	Angle AS	Angle GS
Dziadek	2021	PCL-A2	79.51	81.93
		PCL-A2/1.5PPh	79.51	80.72
		PCL-A2/3.0PPh	75.91	77.12
		PCL-A2/4.5PPh	56.62	66.26

Table S4. Compressive strength values during degradation.

First author	Year	Material	Compressive strength changes in weeks (MPa)					
			0	4	8	12	16	20
Lai	2018	PLGA/TCP-Lai	2.18	1.55	1.60	0.68	0.55	0.40

		PLGA/TCP/ ICA-L	2.28	1.78	1.62	1.53	1.30	0.70
		PLGA/TCP/ ICA-M	2.10	1.70	1.68	1.15	1.15	1.00
		PLGA/TCP/ ICA-H	2.40	1.85	1.78	1.50	1.13	0.73
Chen	2012	PLGA/TCP-Chen	5.40	5.33	4.47	3.61	1.25	
		PLGA/TCP/ LICT	5.78	5.96	5.65	4.71	2.19	
		PLGA/TCP/ MICT	7.20	8.78	6.90	7.05	3.68	
		PLGA/TCP/ HICT	7.88	7.68	6.27	5.56	3.14	
Xie	2010	PLGA/TCP-Xie10	3.79			6.14		
		PLGA/TCP/ ICT-H	3.58			2.86		
		PLGA/TCP/ ICT-M	2.95			1.27		
		PLGA/TCP/ ICT-L	3.37			1.27		

Table S5. Compressive strength changes during degradation.

First author	Year	Material	Strength changes, weeks					
			0	4	8	12	16	20
Lai	2018	PLGA/TCP-Lai	100.00%	71.26%	73.56%	31.03%	25.29%	18.39%
		PLGA/TCP/ ICA-L	104.60%	81.61%	74.48%	70.11%	59.77%	32.18%
		PLGA/TCP/ ICA-M	96.55%	78.16%	77.01%	52.87%	52.87%	45.98%
		PLGA/TCP/ ICA-H	110.34%	85.06%	81.61%	68.97%	51.72%	33.33%
Chen	2012	PLGA/TCP-Chen	100.00%	98.70%	82.78%	66.85%	23.15%	
		PLGA/TCP/ LICT	106.94%	110.37%	104.63%	87.22%	40.56%	
		PLGA/TCP/ MICT	133.33%	162.59%	127.78%	130.56%	68.15%	
		PLGA/TCP/ HICT	145.83%	142.22%	116.11%	102.96%	58.15%	
Xie	2010	PLGA/TCP-Xie10	100.00%			162.05%		

PLGA/TCP/ ICT-H	94.43%	75.48%
PLGA/TCP/ ICT-M	77.78%	33.52%
PLGA/TCP/ ICT-L	88.89%	33.52%

Table S6 Mass changes during degradation

First author	Year	Material	Mass loss over time (days)																							
			0	0.125	1	3	5	7	10	14	20	21	28	30	35	42	49	50	56	60	63	70	77	80	84	90	105
Monavari	2021	ADA-Gel/MSN	100%	76%	81%	75%		69%		49%		36%	34%														
		ADA-Gel/ICA-MSN	100%	80%	62%	73%		63%		43%		38%	36%														
		ADA-Gel/MSN/ICA	100%	84%	63%	77%		64%		45%		36%	35%														
Xie	2019	HA/A	100%		98%			90%		80%		60%	40%		40%												
		HA/A/ICA7	100%		98%			90%		80%		60%	40%		40%												
		HA/A/ICA6	100%		98%			90%		80%		60%	40%		40%												
		HA/A/ICA5	100%		98%			90%		80%		60%	40%		40%												
Lai	2018	PLGA/TCP-Lai	100%					98%		98%		97%	97%		96%		95%				96%		94%		94%		78%
		PLGA/TCP/ICA-L	100%																								82%
		PLGA/TCP/ICA-M	100%																								85%
		PLGA/TCP/ICA-H	100%																								88%
Wang	2013	PLGA/TCP-Wang	100%					98%		97%		95%	94%		93%	92%	91%		89%		87%	84%	82%		80%		
		PLGA/TCP/ICA	100%					98%		97%		97%	96%		95%	94%	94%		92%		91%	91%	90%		89%		
Chen	2012	PLGA/TCP-Chen	100%							96%			92%			89%			85%			84%			80%		
		PLGA/TCP/LICT	100%							98%			95%			92%			91%			89%			86%		
		PLGA/TCP/MICT	100%							98%			96%			94%			92%			91%			86%		
		PLGA/TCP/HICT	100%							99%			98%			96%			93%			92%			91%		

Fan	2012	CS/nHA-Fan	100%				97%		95%					92%	91%			88%		86%		81%		77%		71%
		CS/nHA/ICA-Fan	100%				100%		99%					98%	95%			92%		88%		84%		81%		78%
Xie	2010	PLGA/TCP-Xie10	100%					97%		96%		94%	94%		93%	91%	90%		89%		87%	83%	81%		79%	
		PLGA/TCP/ICT-L	100%					97%		96%		95%	94%		93%	91%	90%		89%		88%	88%	84%		82%	
		PLGA/TCP/ICT-M	100%					98%		97%		96%	95%		95%	94%	94%		91%		90%	90%	89%		89%	
		PLGA/TCP/ICT-H	100%					98%		98%		96%	96%		95%	95%	94%		91%		91%	90%	90%		89%	

Table S7. Changes of pH values during degradation.

				pH changes (weeks)								
First author	Year	Medium	Material	0	2	4	6	8	10	12	14	16
Chen	2012	Milli-Q water	PLGA/TCP-Chen	7.2	6.2	6.0	6.0	5.8	5.7	5.5	5.2	5.1
			PLGA/TCP/LICT	7.2	6.8	6.7	6.6	6.6	6.5	6.3	6.1	6.1
			PLGA/TCP/MICT	7.2	7.1	7.0	6.8	6.8	6.7	6.7	6.6	6.5
			PLGA/TCP/HICT	7.2	7.1	7.0	6.8	6.8	6.7	6.7	6.6	6.5
Xie	2010	PBS with Penicillin/streptomycin	PLGA/TCP-Xie10	7.2	4.6	4.2	3.9	3.9	3.7	3.0		
			PLGA/TCP/ICT-L	7.2	4.6	4.4	4.5	4.2	4.2	3.6		
			PLGA/TCP/ICT-M	7.2	4.9	4.5	4.8	4.6	4.4	4.1		

PLGA/TCP/ICT-L	7.2	5.2	5.2	5.2	4.9	4.8	4.3

Table S8. In vitro release of polyphenols tests - expressed as a percentage.

First author	Medium	Material	Polyphenolic compound	Release of polyphenols (days)																															
				0.125	0.5	1	2	3	4	5	6	7	8	9	10	14	18	20	21	28	30	35	38	40	42	49	50	56	60	63	70	77	80	84	90
Zhao	PBS	SF/HA/0.1NG	Naringin	12.3	46.7	70.0	86.0		90.0																										
Xie	PBS	HA/A/ICA5	Icariin												0.3				0.5		0.7			0.7											
		HA/A/ICA6													0.3				0.4		0.6			0.6											
		HA/A/ICA7													0.2				0.3		0.5			0.5											
Wang	PBS	SF/SBA15IC	Icariin			52.0	64.0	77.0	80.0	82.0	86.0		88.0	90.0	93.0																				
		SF/BMP2/SBA15IC				52.0	64.0	77.0	80.0	82.0	86.0		88.0	90.0	93.0																				
		SF/BMP2/IC/SBA15				94.0	95.0	100.0																											
Pan	PBS	CS/nHS/ICA-Pan	Icariin				18.8			27.1					42.9		51.7			64.6			67.9												
		Magnetic-CS/nHS-ICA					18.8			29.2					29.2		31.3			31.7			31.7												
Wang	SBF	PLGA/TCP/ICA	Icaritin									1.6				5.8			10.9	14.1		15.4			23.0	32.0		42.9		60.0	68.6	72.0		74.6	
Fan	PBS	CS/nHA/ICA-Fan	Icariin							13.9					19.8			25.6			27.9			29.1			31.4		33.7		48.8		62.8		87.2
Xie	SBF	PLGA/TCP/ICT-M	Icaritin									1.2				4.1			9.9	13.3		15.1			17.4	24.9		35.9		49.3	53.3	62.6		71.9	
		PLGA/TCP/ICT-L										1.2				5.8			11.6	14.5		16.2			23.8	32.5		41.7		58.0	66.7	69.0		67.2	
Dziadek	PBS	PCL-A2/1.5PPh	mainly Rosmanic acid		15.6	17.8	16.7	17.8		20.0																									

		PCL-A2/3.0PPh		17.8	18.9	18.9	20.0		20.0						
		PCL-A2/4.5PPh		22.2	37.8	25.6	26.7		27.8						
Cai	α-MEM[1]	mMCS/PK/GE	Genistein	22.5	30.0	41.3	53.8				58.8				73.9

[1] Cell medium α-MEM, 10% fetal bovine serum, 100 U mL^{-1} penicillin and 100 μg mL^{-1} streptomycin sulphate.

Table S9. In vitro release of polyphenols tests - expressed as values.

					Release of polyphenols (days)											
**First author**	**Medium**	**Material**	**Polyphenolic compound**	**Unit**	**0**	**3**	**7**	**14**	**21**	**28**	**35**	**42**	**56**	**70**	**84**	**98**
Monavari	Hanks' Balanced Salt Solution	ADA-Gel/ICA-MSN	Icariin	ug/ml	1.4	2.5	3.2	3.9	4.7	4.8	5.1					
		ADA-Gel/MSN/ICA		ug/ml	1.6	3.0	3.7	4.5	5.8	6.6	6.9					
Lai	10 ml of α-MEM with 1% penicillin and streptomycin	PLGA/TCP/ICA-L	Icariin	ug/10mg			41.4	51.7		55.2		54.6	52.7	53.1	57.3	60.7
		PLGA/TCP/ICA-M		ug/10mg			130.6	157.1		151.3		162.1	183.5	167.1	185.3	191.1
		PLGA/TCP/ICA-H		ug/10mg			94.2	169.7		216.3		304.1	295.7	314.0	349.6	352.7

Table S10 Cell proliferation values.

					The optical density (OD) values (days)						
**First author**	**Year**	**Type of cells**	**Medium**	**Material**	**1**	**2**	**3**	**4**	**5**	**7**	**10**

Yu	2021	Rat bone marrow mesenchymal stem cells (BMSCs)	Not specified	nHA/SF	0.81		1.03			1.21	0.92
				NG/nHA/SF	0.83		1.16			1.22	0.92
				NG/GMs/nHA/SF	0.84		1.18			1.16	0.84
Liang	2021	Bone marrow mesenchymal stem cell	Dulbecco's modified Eagle's medium (DMEM) with 10% fetal bovine serum	HA/SA	0.26		0.71		2.03		
				HA/SA/NG	0.26		1.19		2.29		
Guo	2020	Human mesenchymal stem cells		POC-HA	0.07			0.15		0.18	
				POC-HA/THA	0.09			0.15		0.24	
Xie	2019	Bone marrow mesenchymal stem cell	α-MEM medium	HA/A	0.31	0.49	0.66	0.79			
				HA/A/ICA5	0.40	0.56	0.79	1.10			
Cai	2018	Mouse osteoblast-like MC3T3-E1 cells	α-MEM, 10% fetal bovine serum, 100 U mL^{-1}	mMCS/PK	0.14		0.37			0.51	

			penicillin and 100 μg mL^{-1} streptomycin sulphate								
				mMCS/PK/GE	0.16		0.44			0.58	
Wang	2013	Bone marrow mesenchymal stem cell	Dulbecco's modified Eagle's medium (DMEM)	PLGA/TCP-Wang	0.38		0.60		0.81	0.92	
				PLGA/TCP/ICA	0.53		0.94		1.34	1.39	
Fan	2012	Bone marrow mesenchymal stem cell	Not specified	CS/nHA-Fan	0.18		0.41		0.56	0.72	
				CS/nHA/ICA-Fan	0.18		0.49		0.83	1.39	

Table S11 Cell proliferation changes.

First author, publication year	Type of cells	Medium	Material	The optical density (OD) values changes (days)					
				1	2	3	4	5	7
Yu, 2021	Rat bone marrow mesenchymal stem cells (BMSCs)	Not specified	nHA/SF	100%		127%			149%
			NG/nHA/SF	102%		143%			151%
			NG/GMs/nHA/SF	104%		146%			143%
Liang, 2021	Bone marrow mesenchymal stem cell	Dulbecco's modified Eagle's medium (DMEM) with 10% fetal bovine serum	HA/SA	100%		269%		769%	

		(Gibco, Thermo Fisher Scientific, Inc., USA)							
			HA/SA/NG	100%		451%		867%	
Guo, 2020	Human mesenchymal stem cells		POC-HA	100%			203%		243%
			POC-HA/THA 50/50	125%			203%		324%
Xie, 2019	Bone marrow mesenchymal stem cell	α-MEM medium	HA/A	100%	157%	212%	252%		
			HA/A/ICA5	129%	181%	252%	352%		
Cai, 2018	Mouse osteoblast-like MC3T3-E1 cells	α-MEM, 10% fetal bovine serum, 100 U mL^{-1} penicillin and 100 μg mL^{-1} streptomycin sulphate	mMCS/PK	100%		268%			369%
			mMCS/PK/GE	112%		319%			419%
Wang, 2013	Bone marrow mesenchymal stem cell	Dulbecco's modified Eagle's medium (DMEM)	PLGA/TCP-Wang	100%		155%		211%	239%
			PLGA/TCP/ICA	139%		244%		350%	361%
Fan, 2012	Bone marrow mesenchymal stem cell	Not specified	CS/nHA-Fan	100%		229%		311%	401%
			CS/nHA/ICA-Fan	100%		272%		458%	774%

Table S12 Anti-inflammatory properties.

First author	Cells	Medium	Material	Anti-inflammatory ROS production (RFUs) (days)

				1	3
Dziadek	The murine macrophages (RAW 264.7, ATCC, USA)	Dulbecco's Modified Eagle's Medium containing 10% Fetal Bovine Serum (FBS)	PCL-A2	1.85	1.35
			PCL-A2/1.5PPh	1.68	0.89

Table S13 In vitro antioxidant activity testing.

		RSC ABTS/DPPH (%) and FRAP absorbance (a.u)		
**First author**	**Material**	**ABTS**	**DPPH**	**FRAP**
Dziadek	PCL-A2	1.30	0.00	0.05
	PCL-A2/1.5PPh	2.44	0.00	0.09
	PCL-A2/3PPh	7.75	17.69	0.24
	PCL-A2/4.5PPh	25.48	40.96	0.40

Table S14 ALP activity assay.

					ALP activity osteoblast cells differentation (days)						
**First author**	**Type of cells**	**Medium**	**Materials**	**Unit**	**3**	**5**	**7**	**10**	**14**	**28**	**5-7 days**
Monavari	The undifferentiated preosteoblastic cell line MC3T3-E1	α-MEM[1]	ADA-Gel/MSN	pNpp(nmol/min)			0.01		0.21		100%
			ADA-Gel/ICA-MSN				0.02		0.84		138%
			ADA-Gel/MSN/ICA				0.02		0.76		169%

Dziadek	The Normal Human Osteoblasts (NHOst, Lonza, USA)	OGM[2]	PCL-A2	ALP/cell			5.13		4.04	3.11	100%
			PCL-A2/1.5PPh				6.81		4.63	4.29	133%
Zhao	Human umbilical cord-derived mesenchymal stem cells	Dulbecco's modified Eagle's medium	SF/HA	No specyfied			1.00				100%
			SF/HA/0.03NG				1.29				129%
			SF/HA/0.05NG				1.50				150%
			SF/HA/0.1NG				1.91				191%
Xie	Bone marrow mesenchymal stem cell	α-MEM medium	HA/A	lU/ug			3.75		7.68		100%
			HA/A/ICA5				5.00		9.64		133%
Cai	Mouse osteoblast-like MC3T3-E1 cells	α-MEM[3]	mMCS/PK	OD/mg protein			0.19	0.35	0.57		100%
			mMCS/PK/GE				0.28	0.53	0.73		146%
Wang	Bone marrow mesenchymal stem cell	Dulbecco's modified Eagle's medium	SF/SBA15				0.96				100%
			SF/SBA15IC				1.19				123%
			SF/BMP2/SBA15IC				2.44				254%
			SF/BMP2/IC/SBA15				4.22				439%

Wang	Bone marrow mesenchymal stem cell	Dulbecco's modified Eagle's medium (DMEM)	PLGA/TCP-Wang	IU/mg		20.00					100%
			PLGA/TCP/ICA			28.66					143%
Chen	Bone marrow mesenchymal stem cell	Not specyfied	PLGA/TCP-Chen	IU/mg	12.75		19.78				100%
			PLGA/TCP/LICT		15.82		30.76				156%
			PLGA/TCP/MICT		18.02		37.36				189%
			PLGA/TCP/HICT		14.94		33.84				171%
Fan	Bone marrow mesenchymal stem cell	Not specyfied	CS/nHA-Fan	U/g protein			7.88		14.00		100%
			CS/nHA/ICA-Fan				23.63		37.63		300%

[1] α-modified Eagle's medium (α-MEM), supplemented with 10% (v/v) of Fetal Bovine Serum, 1.0% (v/v) Penicillin-Streptomycin, and 1.0% (v/v) l-Glutamine
[2] Osteoblast growth medium OGM BulletKit containing 10% FBS, 0.1% ascorbic acid and 0.1% GA-1000 (Gentamicin Sulfate and Amphotericin-B)
[3] α-MEM, 10% fetal bovine serum, 100 U mL^{-1} penicillin and 100 µg mL^{-1} streptomycin sulphate
